# What Can Fluorescence Tell Us About Wine?

**DOI:** 10.3390/ijms26073384

**Published:** 2025-04-04

**Authors:** Izabela Sadowska-Bartosz, Grzegorz Bartosz

**Affiliations:** Laboratory of Analytical Biochemistry, Institute of Food Technology and Nutrition, Faculty of Technology and Life Sciences, University of Rzeszow, 4 Zelwerowicza Street, 35-601 Rzeszow, Poland; gbartosz@ur.edu.pl

**Keywords:** wine, fluorescence, front-face fluorescence, chemometrics, PARAFAC, PCA, fluorescent probes

## Abstract

Rapid and cost-effective measurements of the autofluorescence of wine can provide valuable information on the brand, origin, age, and composition of wine and may be helpful for the authentication of wine and detection of forgery. The list of fluorescent components of wines includes flavonoids, phenolic acids, stilbenes, some vitamins, aromatic amino acids, NADH, and Maillard reaction products. Distinguishing between various fluorophores is not simple, and chemometrics are usually employed to analyze the fluorescence spectra of wines. Front-face fluorescence is especially useful in the analysis of wine, obviating the need for sample dilution. Front-face measurements are possible using most plate readers, so they are commonly available. Additionally, the use of fluorescent probes allows for the detection and quantification of specific wine components, such as resveratrol, oxygen, total iron, copper, hydrogen sulfite, and haze-forming proteins. Fluorescence measurements can thus be useful for at least a preliminary rapid evaluation of wine properties.

## 1. Introduction

Wine is one of the oldest and most commonly consumed beverages, consumed since Neolithic times for social, personal, and religious reasons [1,2,3]. Wine is the product of the alcoholic fermentation of grapes, although it is also produced from other fruits. Natural wines contain 9–14% ethanol, while dessert and appetizer wines may contain 15–21% ethanol [4]. The cardioprotective effects of wine have been postulated and debated. The low prevalence of ischemic heart disease, in spite of the high intake of saturated fat, observed in France, was ascribed to the high consumption of red wine and referred to as the French paradox [5,6,7]. Wine is a complex mixture of various compounds including alcohols (mainly ethanol but also small amounts of other alcohols), sugars, esters, aldehydes, ketones, organic acids, amino acids, biogenic amines, furanic compounds, lactones, acetals, phenols, polyphenols, metal ions, terpenes, norisoprenoids, methoxypyrazines, and thiols. All these compounds may affect the taste, aroma, and ither properties of wine and thus consumer preferences [8].

Among the various techniques used to analyze wine, fluorescence spectroscopy is one of less frequently employed. The term “fluorescence” has been introduced to describe the unusual properties of the mineral fluorite, also called fluorspar (calcium fluoride) [9]. Fluorescence denotes a phenomenon occurring in some molecules (fluorophores), consisting of an immediate (in the nanosecond range) emission of light by a substance irradiated with a light of a shorter wavelength or UV radiation. The phenomenon occurs in three stages. In the first step, the fluorophore is excited to an electronic singlet state by absorption of an external photon (*hν*_ex_). In the second step, the excited-state molecule interacts with the molecular environment in several different ways, including vibrational relaxation, quenching, and energy transfer. In the third and final step, a photon (*hν*_em_) of a longer wavelength than that of the exciting light is emitted, while the fluorophore returns to its ground state. The difference between the positions of the band maxima of the absorption and emission spectra is called the Stokes shift [10,11].

Fluorescence spectroscopy offers several advantages over other analytical methods. It is more sensitive than other spectroscopic techniques by 2–3 orders of magnitude [12,13,14,15,16]. In many cases, this technique allows for the rapid assessment of samples without the need for complex preparation processes. Such a measurement is non-destructive and cost-effective. Another approach is based on the use of specific fluorescent probes that can be introduced for the selective measurement of specific compounds or processes, for example, Ca^2+^ [17] or Mn^2+^ concentration [18], the generation of reactive oxygen species [19], or changes in membrane potential [20].

In conventional fluorescence spectroscopy, two types of spectra are usually measured. Recording the emission intensity as a function of the emission wavelength λ_em_ at a fixed excitation wavelength *λ*_ex_ yields an emission spectrum. When *λ*_ex_ is scanned at a fixed *λ*_em_, an excitation spectrum is recorded [10,11,21]. Synchronous spectra are obtained when excitation and emission are scanned simultaneously, with a fixed interval between the excitation and emission wavelengths [22,23].

**Figure 1:**
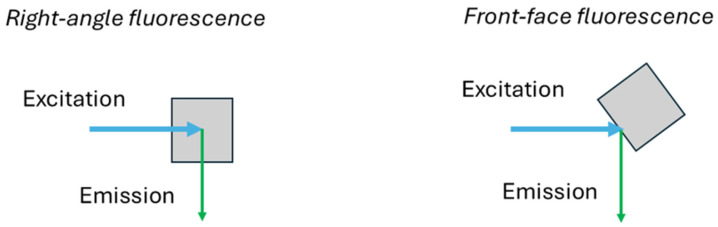
Right-angle and front-face fluorescence.

Like any technique, fluorescence spectroscopy has its limitations. Usually, right-angle measurements of fluorescence are employed. In this arrangement, fluorescence emitted from the whole sample in a plane perpendicular to that of the incident light is measured (Figure 1). However, if the absorbance of the sample at the excitation wavelength is higher than 0.05, the incident light does not reach the whole volume of the sample. In this situation, the intensity of the emitted light may not be accurately measured due to potential scattering and absorption effects. In such cases, it is crucial to dilute the sample or adjust the experimental setup to ensure that the measurements reflect the true fluorescence characteristics of the sample, proportional to fluorophore concentration. This is a common problem when measuring food products, including wine, which are often turbid or opaque or have high concentrations of the fluorophores. However, dilution can lead to the loss of organization of the food matrix. To avoid these problems, the method of front-face fluorescence spectroscopy is used. In this method, fluorescence is emitted from the surface layer of the probe. Thus, excitation of the sample and measurement of its emitted radiation are carried out in the same cell-face. The passage of radiation through the bulk solution is avoided and the scattered light and depolarization phenomena are minimized. The incidence angle of the excitation radiation is between 30° and 60° [10,23,24,25]. Most microplate readers have the option of front-face fluorescence measurement, which may be an incentive for the broader use of this approach. Front-face fluorescence proved to be especially useful in the analysis of wine [26,27,28,29,30].

In principle, the fluorescence signal of a given sample is the sum of the fluorescence contributions from each of the inherent fluorophores. However, fluorescence emission depends on the surroundings of the fluorophore, and in complex systems and concentrated solutions, the fluorescence may not be additive due to the phenomena of quenching, interactions with the molecular environment of the fluorophores, and fluorescence reabsorption [10,11].

## 2. Chemometric Approach to Wine Analysis

**Figure 2:**
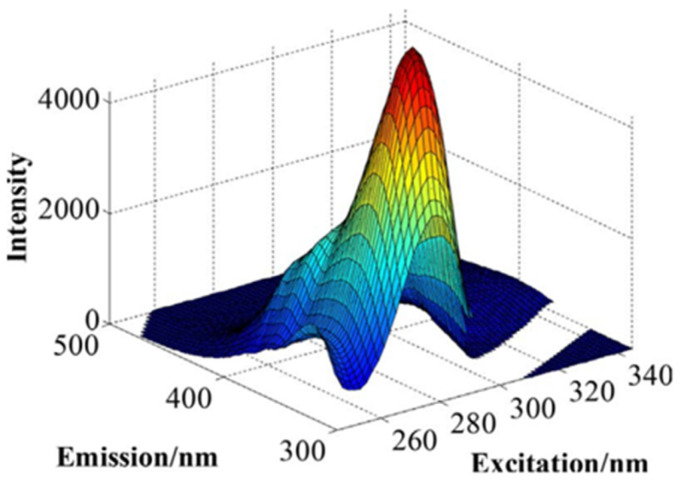
Emission–excitation matrix of a pre-barreled New Zealand wine. From [30], with the kind permission of authors and Elsevier.

In fluorometric studies of complex systems, including wine, a simple emission spectrum for one excitation wavelength is not sufficient to characterize the fluorescent properties of the sample. Instead, a set of emission spectra for a range of different excitation wavelengths *λ*_ex_ is often recorded to obtain a three-dimensional plot, the so-called fluorescence excitation–emission matrix (EEM) (Figure 2).

The excitation–emission matrix can be obtained for each fluorophore. The overall fluorescence EEM for a sample can be described according to Equation (1):

(1) $$EEM=\sum_{i=1}^{n}a_{i}\;\times \;b_{i}\left(\lambda_{ex}\right)\times c_{i}\left(\lambda_{em}\right)$$

where *i* is the number of a fluorophore, *n* is the total number of fluorescent species present in the sample, a*_i_* is a concentration-dependent factor characteristic for each fluorophore, b*_i_*(*λ*_ex_) describes the excitation characteristics, and c*_i_* (*λ*_em_) describes the emission characteristics of the *i*_th_ fluorophore.

The simultaneous presence of multiple fluorophores of interlapping excitation and emission characteristics makes the identification of contributions of individual fluorophores difficult and requires the use of chemometrics, a chemical discipline using mathematics, statistics, and formal logic to analyze the data. Chemometrics uses such tools as parallel factor analysis (PARAFAC), principal component analysis (PCA), or partial least squares (PLS) regression [31,32,33].

The parallel factor analysis (PARAFAC) model is based on a decomposition of a complex set of fluorescence data into several PARAFAC components corresponding to individual fluorophores (or fluorophore groups) present in the samples. In the analysis of data, the relative concentration of components in the mixture can be determined, and the excitation and emission loadings can be used for the identification of fluorophores. The three-way data array is thus decomposed into a set of sample scores, *a_if_*, loadings for the emission mode, *b_jf_*, and loadings for the excitation mode, *c_kf_*. The principle of this approach is to minimize the sum of squares of the residual, e*_ijk_* in Equation (2), using the least-squares algorithm

(2) $$x_{ijk}=\sum_{f=1}^{F}a_{if}b_{jf}c_{kf}+e_{ijk}$$

where *x_ijk_* represents the data for sample *i* in variables *j* and *k* of the two different variable dimensions [12,31].

Principal component analysis (PCA) is another mathematical procedure that decomposes the data matrix with *n* samples and *p* columns (variables, e.g., wavelengths) into the product of a scores matrix, with *n* rows and *d* < *p* columns (principal components, PCs). The scores are the positions of the samples in the space of the principal components, and the loadings are the contributions of the original variables to the PCs. All PCs are mutually orthogonal, and each successive PC contains less of the total variability of the initial dataset. This procedure reduces the dimensionality of the data, which enables the effective visualization, classification, and regression of multivariate data [9]. The PCA components do not necessarily have a clear physical meaning, but they can be efficiently used to understand and classify the wine data. After PCA, data modeling can be further progressed using, e.g., Soft Independent Modeling of Class Analogy (SIMCA) or machine learning as a data modeling alternative [34,35].

Multivariate classification methods or pattern-recognition methods are used for grouping samples with similar characteristics. They include supervised and non-supervised methods.

Non-supervised or exploratory methods can group data into clusters. They are often useful at an early stage of a study to compare subpopulations, such as different batches of a product. Cluster analysis can be performed with simple means, such as hierarchical cluster analysis (HCA) or PCA. HCA compares the similarity between the samples based on their measured variables. The samples are grouped into clusters according to their closeness in a multidimensional space and are usually presented in the form of dendrograms [12]. PCA can also be used to find relationships between different parameters and the detection of possible clusters within the samples [32,36,37].

Sometimes, non-negative matrix factorization (NMF) may be more suitable than PCA. In this method, only positive solution values can be obtained, and thus, this method provides a more realistic approximation to the original data than PCA, which allows for both positive and negative values [38].

In the supervised or discriminant analysis methods, each fluorescence spectrum is preliminarily assigned to a definite class, with comprehensive libraries of spectra representing various versions of each product being constructed in a calibration process. Principal component or partial least squares (PLS) analyses are often applied to spectral datasets to reduce the size of a dataset and co-linearity. Spectral data are analyzed using various methods such as linear discriminant analysis (LDA) [39], factorial discriminate analysis (FDA) [39,40], or k-nearest neighbors (kNN) [41]. The analysis aims at the formulation of weighted linear combinations of the data to minimize the within-class variance and to maximize the between-class variance. If the samples studied are numerous enough, they can be separated into two sets: a training set to elaborate the method (calibration) and a test set to validate it. The elaborated classification rules are later used for allocating new or unknown samples to the most probable subclass [25].

The second stage of analysis is often the factorial discriminate analysis (FDA). This method is useful when the data are preliminarily transformed into their PCs. In the first stage, a stepwise discriminant analysis is performed to select the most relevant PCs for the discrimination of variables when the qualitative classes are initially defined. FDA allows for the construction of new synthetic variables (discriminant factors) from the linear combinations of the selected PCs to achieve a better separation of the centers of gravity of the classes considered. Individual samples are assigned to classes where the distance from the centers of gravity is the shortest. Similarity maps and patterns can be drawn, as in PCA [25,39].

The most frequently used multivariate regression methods for quantitative fluorescence analysis are partial least-squares regression (PLSR) and principal component regression (PCR). Both methods can be used for whole spectra and selected spectral regions, allowing for the inclusion of more information in the calibration model. PCR uses the principal components provided by PCA to perform regression on the sample parameter to be predicted. PLSR points the directions of greatest variability by comparison of the information on both spectral and target properties with the new axes (PLSR components or PLSR factors). The first principal component or factor in PCR represents the widest variations in the spectrum, while in PLSR, it represents the most relevant variations, showing the best correlation with property values of a target [42,43,44].

## 3. Fluorescent Components of Wines

The main fluorescent components of wines are polyphenols. Phenolic compounds are secondary metabolites found in grapes and wine that can be classified into two groups: flavonoids and non-flavonoids (phenolic acids and stilbenes) [45,46]. The phenolic composition of wine is dependent on many factors, including conditions of grape berry development and ripening, the grape cultivar and ripeness at harvest, and the technology of fermentation and aging [47,48].

Within wine flavonoids, three subgroups are important: flavonols, flavan-3-ols, and anthocyanins. Flavonols are found in grape skins as glycosides of myricetin, quercetin, kaempferol, isorhamnetin, syringetin, rutin, and laricitrin [49,50]. Flavan-3-ols (monomeric catechins and polymeric proanthocyanidins) are another large family of polyphenolic compounds comprising mainly catechin, epicatechin, gallocatechin, epigallocatechin, and their corresponding polymers, which are found in the skin and seed of the grape [51,52]. Proanthocyanidins (condensed tannins) are phenolic compounds of a polyflavan-3-ol structure [53,54]. Anthocyanins are extracted from the red grape skins during maceration and fermentation [55,56]. They are highly reactive and easily enter into chemical reactions with other red wine components, such as aldehydes or polyphenols (e.g., tannins), producing new anthocyanin derivatives. Both anthocyanins and anthocyanin-derived pigments contribute to the color of young red wines and play a crucial role in the evolution of wine color during aging [57,58,59,60,61]. Anthocyanin−pyruvic acid adducts appear to be the major anthocyanin derivatives detected by HPLC after only 1 or 2 years of aging in Port wine [58].

Flavonoids are excited in the region of 260–268 nm and emit in the range of 370–422 nm, except for flavan-3-ols which are excited at 278–290 nm and emit in the wavelength range of 310–360 nm [62], although fluorescence at λ_ex_/λ_em_ of 280/310 nm was also reported for catechin [63].

Anthocyanins are weakly fluorescent in solution. A fluorescence quantum yield of 4.1 × 10^−3^ for malvidin 3,5-diglucoside was reported [64], probably due to the efficient excited state proton transfer to water [65]. It may be the main reason why the fluorescence of red wines has been poorly studied. Cyanidin-3-glucoside and malvidin 3,5-diglucoside were reported to have absorption maxima at 220 nm and about 280 nm and fluorescence maxima at 308 and 293 nm, respectively. However, aggregation or complexation to other molecules can induce a significant fluorescence of the resulting anthocyanin-derived compound [66].

Phenolic acids, comprising mainly of caftaric, coutaric, fertaric, and tartaric, are normally found as esters. Hydroxycinnamic esters are one of the most abundant groups of phenolic compounds found in grapes. Stilbene-like compounds include resveratrol, its glucoside piceid, astringin, and viniferins [67,68]. The excitation wavelengths of phenolic acids (both derivatives of cinnamic acid and derivatives of benzoic acid), phenolic aldehydes, and stilbene-like compounds extend between 260 nm and 330 nm, while the emission wave range is 320–440 nm [62].

Other fluorescent molecules present in wine, apart from polyphenols, are vitamins and amino acids. Vitamin A (retinol) is present in wine in very small amounts. The excitation maximum corresponds to about 335 nm, and the emission maximum to about 470 nm [69]. On the other hand, B-complex vitamins are the most abundant. Riboflavin is present mainly as a component of flavin-mononucleotide (FMN) and flavin-adenine-dinucleotide (FAD). Free riboflavin is also present in raw and processed fruits and is present in significant amounts in wine. Flavin absorption is centered at about 450 nm and emission at about 525 nm [70]. The fluorescent amino acid tryptophan and its ethyl ester have been reported to be present in wines [71,72,73]. Tryptophan is excited at wavelengths around 280 nm and emits fluorescence in the range of 300–400 nm [27,71]. NADH is formed in the fermentation processes that take place during the production of wines [74]. NADH is excited at 340–350 nm and emits fluorescence centered at 460–470 nm [75]. Fluorescent oxidation products and Maillard products may be produced in the browning processes during the aging and storage of wines [76]. Due to their heterogeneity, the absorption maxima of Millard reaction products may range from 320 to 450 nm, while emission maxima are in a broad range of 380–530 nm [77].

## 4. Analysis of Wine Autofluorescence

An example of an EEM of wine constructed by the measurement of a series of fluorescence spectra for a range of excitation wavelengths is shown in Figure 2. The list of components tentatively assigned to four PARAFAC components used to interpret EEM of this type is shown in Table 1.

Analysis of these EEMs by PARAFAC makes it possible to determine the relative contributions of each component to the spectra matrices. Other studies found that red wines have four main fluorescence components, with the excitation and emission maxima at the wavelength pairs of 260/380 nm, 275/323 nm, 330/410 nm, and 280/364 nm, respectively [27,78]. A tentative identification of fluorophores performed by matching PARAFAC score values with the HPLC analysis of wine revealed that the third component correlated with concentrations of catechin and epicatechin [30,36]. Such measurements allowed for distinguishing between Rioja and Ribera del Guadiana wines, discrimination between Rioja and non-Rioja samples, discrimination between Crianza or Reserva wines compared to young wines [27], and discrimination of wines according to the country of origin [78].

Components tentatively assigned to four PARAFAC components used for the analysis of Cava wines are shown in Table 2. The fluorescence analysis of sparkling cava wines was found to be a fast alternative method for the quality control of sparkling wines. Specifically, monitoring the fluorophores centered at excitation/emission of 465/530 nm and 280/380 nm can provide useful information about the chemical changes occurring during browning [76].

Pre-barreled New Zealand Pinot Noir wines showed an EEM with a component characterized by excitation and emission maxima at around 277 nm and 330 nm, respectively. The maximum signal intensity of this component was increased about two times in comparison with the grape juice. This increase is contributed by multiple fluorophores in the wine, such as the phenolic acids (syringic, vanillic, gallic, caftaric, *p*-hydroxybenzoic and caffeic acid, catechin, epicatechin and tryptophan) and other components that were not present or present at much lower levels in grape juice [30].

In the study of red wines by dos Santos et al., the total phenolics region corresponded to 260–360 nm excitation and 370–400 nm emission, the total condensed tannins were the main contributor to the fluorescence in the region of excitation between 285 and 340 nm and emission in the range of 290–350 nm, and the total anthocyanins region contributed to the signals with excitation between 280 and 300 nm and emission between 330 and 380 nm [79]. The analysis of EEMs of 200-times diluted Cabernet Sauvignon wines from three regions of Australia and Bordeaux using discriminant analysis and support vector machine discriminant analysis (SVMDA) made it possible to differentiate wines according to the location of origin [80]. The analysis of EEMs of 150-times diluted Shiraz, Cabernet Sauvignon, and Merlot wines from 10 locations in Australia by PLS and extreme gradient boosting (XGB) discriminant analysis (a machine learning protocol) allowed for the differentiation of samples by their variety and geographical origin [81].

The analysis of wine phenolic content by front-face fluorescence spectroscopy combined with chemometrics was suggested to be a potentially useful tool for authentication and quality control by regulatory bodies [33]. The use of principal component analysis (PCA) and classification by factorial discriminant analysis (FDA) allowed for distinguishing between German and French wines, demonstrating the possibility of identification of wines according to variety and typicality [26]. Discrimination between Shiraz, Cabernet Sauvignon, and Pinot Noir based on fluorescence can be improved by measurements at different pH levels [28].

Front-face fluorescence spectroscopy in combination with PARAFAC was also shown to be a promising tool for the discrimination of grape-derived products from different clonal and vineyard site origins within a small geographical region in New Zealand. The discrimination between grape clones was found to be due to higher concentrations of the component at an excitation maximum of 260 nm and emission maximum of 390 nm, with a shoulder at 370 nm, possibly contributed by caffeic acid-related fluorophores. The effect of discrimination based on the vineyard site was indicated to be due to the component at an excitation maximum of 278 nm and emission maximum of 360 nm, probably contributed mainly by tryptophan and hydroxylated benzoic acid derivatives [30].

The fluorescence of bulk Slovak Tokaj wines was characterized by an excitation range of 390 to 500 nm, with a maximum at 460 nm and emission in the range of 450 to 590 nm, with a maximum of about 530 nm. These wines, when diluted 500 times, had fluorescence characterized by excitation in the range of 250–350 nm and emission in the range of 320–450 nm. An intense band was observed with excitation in the range of 270–280 nm and emission centered at 350 nm, as well as a weak band with excitation at 300–310 and emission at about 430–440 nm. This fluorescence was similar to those of phenolic acids characterized by excitation/emission wavelengths: (gallic acid, 280/360 nm; protocatechuic acid, 270/350 nm; caffeic acid, 262 and 325/426 nm; caftaric acid, 290 and 325/440 nm, *p*-coumaric acid 290 and 309/404 nm) and catechin, 280/310 nm. The PLS regression allowed for the estimation of the content of gallic, protocatechuic, caffeic, and *p*-coumaric acids, and (+)-catechin from the fluorescence spectra of wines [63].

Synchronous spectra in the range of 260–290 nm, especially with the wavelength difference between excitation and emission (Δ*λ*) of 60 to 100 nm, allow for prediction of the antioxidant capacity of wines based on the estimation of the concentrations of phenolic compounds in sweet Slovak Tokaj wines [82]. Emission spectra corresponding to excitation at 320 nm or synchronous spectra in the range of 300–400 nm, especially with Δλ of 80 nm, allow for the determination of the sum of concentrations of coumarins in Tokaj wines [83].

A Port red wine aged 3 years showed a fluorescence maximum at about 595 nm when excited at 500 nm. The fluorescence maximum was shifted by about 30 nm towards shorter wavelengths concerning the young wine (fluorescence maximum at about 625 nm). Aged wines usually contain larger amounts of polymerized anthocyanin (pol-Anth) pigments and lower amounts of monomeric anthocyanin pigments than young wines. The fluorescence band of pol-Anth isolated from wine and dissolved in 12% ethanol brought to pH 3.3 was similar to that of aged wines (peak at 597 nm), while the spectra of mon-Anth showed a bathochromic shift of about 40 nm in comparison with pol-Anth. The intensity of fluorescence was higher for pol-Anth than mon-Anth [84].

The fluorescence intensity ratio at 700 nm to that of 560 nm of wines excited at 500 nm was found to decrease when plotted against the relative share of pol-Anth and to decrease in old wines, and it was proposed to be a measure of the pol-Anth/min-Anth ratio, and thus of red wine age.

The treatment of young red wine with sulfur dioxide caused a hypsochromic shift in the fluorescence spectrum to match the spectrum of old Port wine. This effect is due to the formation of colorless and nonfluorescent compounds in the reaction of monomeric anthocyanins with sulfur dioxide, which binds to carbon 4 of the C ring. Polymerized anthocyanins remain unbleached because the site of sulfite binding is the same as that engaged in the anthocyanin polymerization [84,85].

Wine anthocyanins react with pyruvic acid, forming pyranocyanins such as vitisin A [86,87]. It should be noted that the term “vitisin A” is ambiguous since the same name is used for another compound, one of resveratrol tetramers [88,89]. Malvidin 3-*O*-glucoside (Mv 3-*O*-glc) is the major anthocyanin detected in young red wine and vitisin A (a pyruvic adduct of Mv 3-*O*-glc) [58]. The fluorescence spectrum of Mv 3-O-glc at pH 1.0 showed a peak at 610 nm, which increased in intensity and shifted to 638 nm at pH 3.3. Vitisin A showed a broad emission band with a maximum around 720 nm at pH 1.0, while at pH 3.3, a significant increase in fluorescence was observed around 630 nm. The fluorescence quantum yield of vitisin A relative to Mv 3-O-glc was 0.81 and 0.86 at pH 1 and 3.3, respectively. The increase in the fluorescence intensity, for both Mv 3-O-glc and vitisin A, from more to less acidic conditions can be explained by proton quenching, the extent of which is pH-dependent. Based on differences in the excitation spectra, the fluorescence excitation ratio (FER) between wavelengths at the maximal difference and the isosbestic point (FER_550nm/425nm_ and FER_350nm/425nm_) was proposed to estimate the relative amounts of Mv 3-*O*-glc and vitisin A, although in whole wines, the strong contribution of pol-Anth to fluorescence makes it difficult to distinguish the two classes of pigments [84].

One technique of measuring the fluorescence of dye molecules concentrated on the surface of wine as a difference between fluorescence was proposed based on the estimation of the difference between the fluorescence from the surface and from inside the wine [90].

Simple measurements of wine fluorescence may not require a spectrofluorimeter or plate reader. The fluorescence spectrum of a red wine from the Lazio region obtained by a computer screen photo-assisted technique (a combination of computer monitors and webcams) was characterized by an excitation maximum at 450 nm, a shoulder at 520 nm, and an emission maximum at about 610 nm [91].

## 5. Application of Fluorescent Probes for the Analysis of Wine

The use of fluorescent probes allows for the selective estimation of the content of chosen parameters of components of wine using a spectrofluorimeter, plate reader, or fluorescence microscope. The applications of flow cytometry for the analysis of wine are not included in this review, as they require specialized equipment.

A fluorescence-based sensor based on the measurement of the luminescent lifetime of a reference metal/porphyrin complex was proposed for the real-time monitoring of oxygen concentration during wine fermentation. The sensor allows for the determination of the concentrations of O_2_ in wine below 10–40 µg/L at 20 °C, i.e., between 0.1 and 0.5% O_2_ [92]. The precise determination of oxygen content during wine production is important, as oxygen induces changes in the chemical and sensory profile of wines, including the final alcohol content. Moderate red wine exposure to oxygen has a positive impact on the color, aroma, and taste of red wine’s properties. However, oxygen negatively affects the quality and sensory properties of white wines [93,94].

A fluorescence assay for resveratrol determination based on Förster Resonance Energy Transfer (FRET), via competitive supramolecular recognition, between *p*-sulfonated calix [6] arene-(CX6)-modified reduced graphene oxide (CX6@RGO) and Rhodamine B- or rhodamine 123–resveratrol complex was explored. Resveratrol present in wine competes with the complex, releasing rhodamine and causing an increase in the fluorescence intensity. This assay, not requiring any wine pretreatment, allows for the determination of resveratrol with a detection limit of 0.5 µM [95].

The off-odors of wine are of considerable concern in the wine industry. They can develop after the wine has been bottled, and no corrective action is possible. Hydrogen disulfide is the main component of wine off-odors, which are also contributed by mercaptans and volatile sulfur compounds [96,97]. A fluorescent probe, 4-methyl-2-oxo-2H-chromen-7-yl-thiophene-2-carboxylate, was used to estimate the level of hydrogen sulfide in wine. Three red wines bought in a Beijing supermarket were found to contain 0.48, 0.45, and 0.55 μM H_2_S when estimated using this probe [98].

A fluorescent probe (E)-2-((4-(benzo[d]thiazol-2-yl)benzylidene)amino)phenol (BTPAP) was applied for the determination of total iron (Fe^2+^/Fe^3+^) in wine. The proposed assay based on the probe allows for the estimation of iron with a limit of detection of 1.16 µM and a linearity of response up to 200 µM. In three samples of Chinese red wines, the iron concentration was found to be 24–37 µM with this probe [99].

At least three fluorescent probes have been applied for estimation of the copper level in wines. The application of a fluorescent coumarin-based probe for the detection of copper(II) in wine revealed Cu^2+^ concentrations of 0.22–0.46 in three red wines. The detection limit of the method was 62 nm, and the range of linearity was 0–16 µM [100]. A pH-sensitive chemosensor based on Rhodamine B coupled to a tetraazamacrocyclic ring, 3 [N-(9-(2-((1,4,7,10-tetraazacyclotridecan-5-yl)methyl)-3-oxoisoindolin-1-yl)-6-(diethylamino)-3H-xanthen-3-ylidene)-*N*-ethylethanamine], was used to determine the Cu^2+^ concentration in white wine. The detection limit of the sensor is 43.8 nM. The Cu^2+^ concentration in six samples of a 2016 Burgundy Chardonnay was found to be 0.03–0.76 mg/L (0.47–11.96 µM) [101]. A fluorometric assay based on the simultaneous use of two fluorescent probes and the measurement of the ratio of fluorescence intensities at two wavelengths allows for the determination of the copper concentration in wine, with a limit of detection of 46.5 nM and a linear range up to 4 µM. The Cu^2+^ concentrations in three samples of red wine were found to be 22–36 µg/L (0.35–0.57 µM) [102].

Ochratoxin A is a hepatotoxic, genotoxic, cytotoxic, and teratogenic mycotoxin produced by several fungal species, mainly of the genera *Aspergillus* and *Penicillium*, that can contaminate wine. A fluorescence polarization immunoassay for ochratoxin A possesses a detection limit of 0.11±0.05 ng/mL [103].

Protein haze is an esthetic problem in white wines caused by the persistence of grape pathogenesis-related proteins that are highly stable during winemaking. Some of these proteins precipitate over time, especially at elevated temperatures, forming a turbid haze [104,105]. A rapid fluorescence-based technology to detect haze-forming proteins in white wines was developed based on the use of a new fluorescent probe binding selectively to haze-forming proteins. The method has a detection limit of 2 mg/L and a linear range of 4 to 400 mg/L. It is sensitive enough, since the minimal concentration of proteins to form haze is 12 mg/L [106].

Staining lactic acid bacteria with the Live/Dead BacLight^TM^ staining kit and fluorescent microscopy assessment allows for control of the viability of lactic acid bacteria and thus the process of malolactic fermentation used for the biological deacidification of wines [107].

Examples of the application of fluorescence measurements to wine are listed in Table 3.

Wine has been one of the most popular drinks worldwide for thousands of years. Monitoring wine quality and controlling forgeries concerning the false reporting of wine origin and quality is important. Fluorescence spectroscopy, allowing for a rapid estimation of a range of wine properties, requiring only a fluorimeter or plate reader, and available in most laboratories, may be very useful in this respect, especially for preliminary wine screening.

## Figures and Tables

**Table 1:** Tentative assignment of fluorophores to fluorescence components identified by PARAFAC in New Zealand Pinot Noir wines, following [30].

Component	*λ*_max_ (exc)	*λ*_max_ (em)	Tentatively Assigned Fluorophores
1	278	315	Monomeric catechins
2	278	360	Tryptophan, vanillic acid, syringic acid, gallic acid
3	260	(370)/ 390	Caffeic acid
4	(278)/ 320	415	Caffeic acid, p-coumaric acid, tyrosol

**Table 2:** Fluorescence components tentatively identified by PARAFAC in cava wines, and tentative assignment of fluorophores. After [76].

Component	*λ*_max_ (exc)	*λ*_max_ (em)	Tentatively Assigned Fluorophores
1	395	485	Unknown
2	365	440	Oxidation products, Maillard products, NADH
3	465	530	Vitamin B_2_ or riboflavin
4	280, shoulder at 350	415	Stilbenes such as *trans*-piceid and *trans*-resveratrol

**Table 3:** Results of fluorescence analysis of wine.

Wine	Analysis	Results	Reference
French and German wines	Front phase, PCA	Differentiation between Gamay and Dornfelder wines, discrimination between typical and non-typical Beaujolais wines	[26]
Red wines	PARAFAC	Discrimination according to the country of origin and grape variety	[78]
Red wines	Front phase, PARAFAC	Separation between Rioja and Ribera del Guadiana wines, discrimination between Rioja and non-Rioja samples for Crianza and Reserva wines compared to young wines	[27]
White Argentinian wines	PCA, PARAFAC, other algorithms; best results with U-PLS-DA	Discrimination between the type of grape used for wine production	[108]
New Zealand Pinot Noir	Front phase, PARAFAC	Detection of differences in vineyard site, grape clone, winemaking process, and barrel properties	[30]
South African red wines	Front phase, PARAFAC, PCA, Bayesian optimization	Classification of South African red wine cultivars based on unique fluorescent fingerprints	[33]
White wines	PCA-LDA	Discrimination between Furmint, Lipovina, and Muscat Blanc wines	[109]
Pinot Gris and Riesling wines (Romania), Riesling (Romania) and Sauvignon (France)	Classical right-angle fluorimetry, PARAFAC, SIMCA	Classification based on the site of origin	[110]
Cabernet Sauvignon wines from 3 regions of Australia and Bordeaux	EEM of 200 times diluted wines analyzed by DA and SVMDA	Discrimination of wines according to location	[80]
Shiraz, Cabernet Sauvignon, and Merlot wines from 10 locations in Australia	EEM of 150-times diluted wines analyzed by XGB discriminant analysis and PLS	Discrimination of wine brand and geographical location	[81]
Four- to six-butt Tokaj wines	PCA followed by LDA	Distinguishing between botrytized wines of different quality (4-, 5- and 6- butt wines) and between unadulterated and adulterated wines	[111]
Cava sparkling wines	PARAFAC	Monitoring of browning in sparkling wines	[76]
Ribera del Guadiana and Rioja wines	Front phase, U-PLS/RBL	Good results for the quantification of caffeic and vanillic acids and resveratrol; acceptable results for epicatechin	[112]
Red wines (Cabernet Sauvignon)	Front-phase fluorescence; PCA, RMSE and MAE	Estimation of the content of total phenolics, total condensed tannins, and total anthocyanins following the course of fermentation	[79]
White Chardonnay wines	PARAFAC	Detection of the effect of SO_2_ treatment and/or vintage, even after several years of bottle aging	[113]
Porto wines and table red wines, Portugal	Diluted wines, standard fluorescence spectra	Fluorescence F_700nm_/_F560nm_ ratio as a measure of monomeric/polymeric anthocyanins; excitation ratio F_ex350nm_/F_ex 550 ratio_ as a measure of vitisin A/malvidin-3-*O*-glucoside ratio	[84]
Sweet Tokay wines	Synchronous emission spectra (260–290 nm), Δ*λ* of 60 to 100 nm	Prediction of antioxidant capacity of wines based on estimation of the concentrations of phenolic compounds	[82]
Tokaj wines	Spectra at *λ*_ex_ = 320 nm or synchronous fluorescence spectra	Determination of sum of concentrations of coumarins	[83]
Tokaj wines	Bulk and diluted (500 times), PLS	Estimation of concentrations of gallic, protocatechuic, caffeic, and *p*-coumaric acids and (+) catechin	[63]
White and red wines	Fluorescence sensor	Estimation of oxygen level in wine	[92]
Red wines	FRET-based fluorescence assay	Estimation of resveratrol concentration in wine	[95]
Red wines	Fluorescent probe 4-methyl-2-oxo-2H-chromen-7-yl-thiophene-2-carboxylate	Estimation of the level of H_2_S in wine	[98]
Red wine	BTPAP fluorescent probe	Estimation of Fe^2+^/Fe^3+^ concentration in wine	[99]
Red wine	Coumarin-based fluorescent probe	Estimation of Cu^2+^ concentration in wine	[100]
White wine	Macrocyclic Rhodamine B-based fluorescent probe	Estimation of Cu^2+^ concentration in wine	[101]
Red wines	Simultaneous use of two fluorescent probes, fluorescence intensity ratio at two wavelengths	Estimation of Cu^2+^ concentration in wine	[102]
White wines	New fluorescent probe	Estimation of concentration of haze-forming proteins	[106]
Red wines	Fluorescence polarization	Immunoassay for ochratoxin	[103]

DA, discriminant analysis; MAE, mean absolute; PLS, partial least squares regression; RMSE, root mean square error; SIMCA, Soft Independent Modeling Classification Analogy; SVMDA, support vector machine discriminant analysis; U-PLS/RBL, unfolded-partial least squares coupled to residual bilinearization; XGB, extreme gradient boosting.

## Data Availability

Data will be available from the corresponding author upon reasonable request.
